# Nuclear localization of a *C. elegans* CCCH-type zinc finger protein encoded by T26A8.4

**DOI:** 10.17912/YHG7-JE66

**Published:** 2018-09-29

**Authors:** Takashi Koyama, Chisato Ushida

**Affiliations:** 1 Functional Genomics and Biotechnology, United Graduate School of Agricultural Science, Iwate University, 18-8 Ueda 3-chome, Morioka 020-8550; 2 Department of Biochemistry and Molecular Biology, Faculty of Agriculture and Life Science, Hirosaki University, 3 Bunkyo-cho, Hirosaki, 036-8561

**Figure 1.**
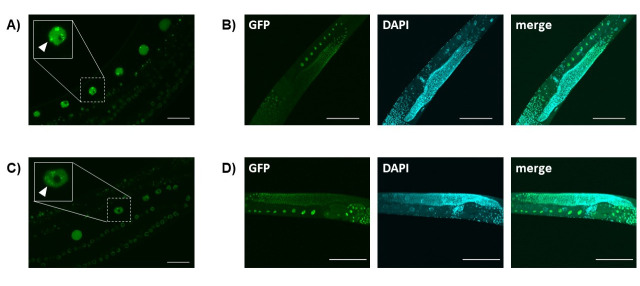


## Description

T26A8.4 encodes a CCCH-type zinc finger protein. The amino acid sequence of this protein is partially similar to that of *S. cerevisiae* Caf120p, which has no zinc finger domain. T26A8.4-encoded RNA was detected more abundantly in germ cells than in other tissues of L4-adult worms according to the NEXTDB data. When expressed specifically in the germline under control of the *mex-5* promoter, the T26A8.4-encoded protein localized to germ cell nuclei in the adult hermaphrodite and can be seen in foci (arrowheads in the insets of panels A and C). During germline development, gene expression is primarily regulated posttranscriptionally by the mRNA stability and/or translation with the 3’UTRs (Merritt *et al*. 2008). To test the post-transcriptional regulation of T26A8.4 expression, T26A8.4::GFP with its own 3’UTR and that with *tbb-2* 3’UTR were compared. The results showed that T26A8.4 is expressed throughout the germline with both 3’UTRs, indicating that T26A8.4 does not appear to be post-transcriptionally regulated by its 3’UTR.

pTKD841 was made by cloning a fusion of PCR fragments of genomic *mex-5* promoter with *mex-5*5’UTR (523 bp), genomic T26A8.4 (2068 bp), a linker amino acid (Gly-Gly-Gly-Gly-Gly-Ala) coding sequence (18 bp), *gfp* (870 bp) and genomic T26A8.4 3’UTR (459 bp) with its downstream sequence (144 bp) into the Sbf1 site of a plasmid vector pCFJ151 (Zeiser *et al*. 2011). pTKD842 was made as pTKD841 except for the 3’UTR. Genomic *tbb-2* 3’UTR (297 bp) with its downstream sequence (32 bp) was fused to *gfp* in pTKD842. These plasmids were introduced into *C. elegans* EG4322 with plasmids pRF4 and pCFJ601 to make strains HUJ0001 and HUJ0002, respectively, by a MosSCI method (Frøkjær-Jensen *et al*. 2008; Frøkjær-Jensen *et al*. 2012). GFP signal was detected in the germ cell nuclei in both strains. Panels A) and C) show GFP signal from live imaging of HUJ0001 and HUJ0002, respectively. The inset of each panel shows the magnified image of an oocyte (dashed square) in an adult hermaphrodite. The foci were observed in the nucleus of diplotene stage to that of -1 oocyte. Some pachytene stage nuclei also exhibited the foci. Scale bars, 20 mm. Panels B) and D) show GFP signal with DAPI staining image of the fixed specimens of HUJ0001 and HUJ0002, respectively. The foci observed from the live imaging could not be detected. GFP, T26A8.4::GFP; DAPI, DAPI staining image; merged, merged image of GFP and DAPI. Scale bars, 100 um.

## Reagents

Plasmids:

pCFJ151 (*ttTi5605_MCS*)

pCFJ601 (*Peft-3::Mos1 transposase::tbb-2 3’UTR*)

pRF4 (*rol-6 (su1006)*)

pTKD841 (*Pmex-5::mex-5 5’ UTR::T26A8.4::GFP::T26A8.4 3’ UTR*)

pTKD842 (*Pmex-5::mex-5 5’ UTR::T26A8.4::GFP::tbb-2 3’ UTR*)

*C. elegans* strains:

EG4322 (*ttTi5605mos II; unc-119 (ed3) III*)

HUJ0001 (*huaIs001*[pTKD841(*Pmex-5::mex-5 5’ UTR::T26A8.4::GFP::T26A8.4 3’ UTR Cbr-unc-119 (+))] II; unc-119 (ed3) III*)

HUJ0002 (*huaIs002*[pTKD842(*Pmex-5::mex-5 5’ UTR::T26A8.4::GFP::tbb-2 3’ UTR Cbr-unc-119 (+))] II; unc-119 (ed3) III*).
